# Incisional Hernia After Minimally Invasive Radical Prostatectomy: A Systematic Review

**DOI:** 10.1016/j.euros.2026.08.002

**Published:** 2026-09-28

**Authors:** Arthur Peyrottes, Olivier Oberlin, Michael Baboudjian, Charles Dariane, Frederic Panthier, Pierre Mongiat-Artus, François Desgrandchamps, Guillaume Ploussard, Alexandra Masson-Lecomte

**Affiliations:** a Department of Urology, Hôpital Saint-Louis, AP-HP, Université Paris Cité, Paris, France; b Department of General Surgery, Groupe Hospitalier Privé Ambroise-Paré - Hartmann, Paris, France; c Department of Urology, Hôpital Nord, Université Aix-Marseille, Marseille, France; d Aix Marseille Univ, CNRS, CINAM, ERL INSERM U 1326, CERIMED, Marseille, France; e Department of Urology, Hôpital Européen Georges Pompidou, AP-HP, Université Paris Cité, Paris, France; f Paris University, U1151 Inserm-INEM, Necker, Paris, France; g Department of Urology, Hôpital Tenon, AP-HP, Sorbonne Université, Paris, France; h Department of Urology, La Croix du Sud Hospital, Quint Fonsegrives, France

**Keywords:** Prostate cancer, Radical prostatectomy, Incisional hernia, Pfannenstiel incision, Systematic review

## Abstract

**Purpose:**

Incisional hernia (IH) is an under-recognized complication after minimally invasive radical prostatectomy (MIRP).

**Methods:**

We performed a systematic review according to Preferred Reporting Items for Systematic reviews and Meta-Analyses guidelines to evaluate the proportion, risk factors, and surgical determinants of IH after MIRP.

**Results:**

Sixteen studies published between 2010 and 2025 were included, encompassing 56 661 patients. Most were retrospective cohorts, with heterogeneous definitions and diagnostic methods. The reported proportion of IH ranged from 0.04% to 31%, with a median time to diagnosis of 14 mo. Repair was required in up to 80% of cases, and emergency repair in up to 15%. Two comparative studies directly evaluating specimen extraction site consistently reported lower proportions of IH with transverse suprapubic (Pfannenstiel) extraction than with periumbilical extraction, while three registry-based studies similarly reported lower proportions following open compared with MIRP. Evidence regarding the influence of fascial incision orientation on reported IH occurrence was mixed, whereas no clear difference was reported between midline and off-midline extraction sites. Reported factors associated with IH included advanced age, obesity, larger prostate volume, postoperative parietal abscess, and prior abdominal surgery. Limitations include the heterogeneity in the studies included.

**Conclusions:**

These findings highlight IH as a clinically meaningful complication of MIRP. Reported IH occurrence differed by specimen extraction site in the available observational studies, but the evidence remains limited by heterogeneous definitions, follow-up, and risk of bias. Preventive strategies, including the site of specimen extraction, warrant further prospective evaluation.

## Take Home Message

Incisional hernia is a frequent yet under-recognized complication of minimally invasive radical prostatectomy. Our systematic review highlights the extraction site as a key determinant, with Pfannenstiel incisions showing lower hernia rates than supraumbilical approaches.

ADVANCING PRACTICE **What does this study add?**

Incisional hernia is an under-recognized complication after minimally invasive radical prostatectomy, with reported proportions ranging widely according to definitions and ascertainment methods. This systematic review highlights substantial heterogeneity in the evidence but identifies specimen extraction site as a potentially modifiable determinant, with lower reported hernia proportions following suprapubic than periumbilical extraction. These findings support prospective evaluation of extraction strategies and standardized follow-up to better define the burden of incisional hernia and strategies for its prevention.

**Clinical Relevance**

Incisional hernia is a clinically meaningful but potentially under-recognized complication after minimally invasive radical prostatectomy, sometimes requiring surgical and emergency repair. Current evidence suggests that specimen extraction through a transverse suprapubic (Pfannenstiel) incision may reduce the risk of incisional hernia compared with periumbilical extraction, although the available evidence remains observational and heterogeneous. Awareness of this complication and prospective evaluation of modifiable surgical factors could help inform preventive strategies and improve long-term outcomes after minimally invasive radical prostatectomy. Associate Editor: Roderick C.N. van den Bergh.

**Patient Summary**

Hernias in the abdominal wall can occur after prostate cancer surgery, particularly after minimally invasive procedures. Some studies suggest that the reported occurrence of hernias may differ according to the site used to remove the prostate, with lower reported proportions after transverse suprapubic (Pfannenstiel) extraction than after periumbilical extraction. However, most of the available evidence comes from retrospective studies with heterogeneous follow-up, and further prospective studies are needed to determine whether changing the specimen extraction site reduces clinically relevant hernias.

## 1 Introduction

Radical prostatectomy (RP) remains a standard curative treatment for localized prostate cancer. Minimally invasive radical prostatectomy (MIRP), including laparoscopic and robot-assisted techniques, has progressively replaced open retropubic approaches owing to reduced blood loss, shorter hospital stay, and faster postoperative recovery [1]. However, as perioperative morbidity has decreased, attention has shifted toward later complications, including incisional hernia (IH). Although traditionally considered uncommon, IH after MIRP is increasingly recognized in clinical practice and population-level data [2]. IH may impair patients’ quality of life, necessitate abdominal wall reconstruction, and in some cases lead to emergency surgery.

Despite these implications, IH remains underreported in urological literature for several reasons. First, definitions of IH vary widely (from clinical examination to patient-reported outcomes to systematic imaging), yielding inconsistent detection proportions [3]. Second, follow-up after RP is primarily oncologic and urologic; abdominal wall evaluation is rarely standardized, and patients often undergo hernia repair in other departments or institutions, resulting in incomplete capture of events. Third, heterogeneity in surgical technique contributes to variability: specimen extraction has been performed through supraumbilical camera ports, infraumbilical midline incisions, off-midline ports, and suprapubic incisions, each associated with different fascial orientations and closure constraints. This diversity complicates comparisons across studies and limits the interpretability of individual series.

Given this heterogeneity, the true proportion and determinants of IH after MIRP remain uncertain. Importantly, the extraction site and the manner in which the fascia is incised and closed may be modifiable contributors to hernia development, yet evidence has never been systematically synthesized. To address these gaps, we conducted a systematic review to evaluate the proportion, risk factors, and surgical determinants of IH after MIRP, with a specific focus on the impact of specimen extraction site.

## 2 Materials and methods

This systematic review was conducted according to the Preferred Reporting Items for Systematic reviews and Meta-Analyses guidelines [4]. A comprehensive search of PubMed, Embase, and Web of Science was performed from database inception to October 2025, combining the terms “radical prostatectomy”, “incisional hernia”, “parietal hernia”, and “eventration”. A protocol was registered in the International Prospective Register of Systematic Reviews database (registration number: CRD420251156270). Eligibility criteria included English-language studies reporting the proportion of IH (Outcome) in patients treated with MIRP (pure laparoscopic or robot-assisted, Intervention) for localized prostate cancer (Population). Comparative studies reporting IH proportions across surgical approaches or extraction techniques (Comparator) were also included. Additional data extracted included study characteristics, definition and ascertainment method of IH, duration of follow-up, surgical technique, specimen extraction site, fascial closure technique, and reported patient- and surgery-related risk factors. When available, adjusted effect estimates from multivariable analyses identifying predictors of IH were also collected. Exclusion criteria were nonoriginal articles or studies not providing extractable data on IH.

Two reviewers (A.P., A.M.L.) independently screened titles, abstracts, and full texts. Discrepancies were resolved by consensus with a third party (G.P.).

The primary end point was the proportion of IH according to individual study definition. Secondary end points included time to diagnosis, repair proportion, extraction technique, fascial closure, and patient- or surgery-related risk factors. The studies’ quality and risk of bias (RoB) were independently assessed using the ‘‘Risk Of Bias In Non-randomized Studies - of Interventions (ROBINS-I)’’ tool [5] and the “Revised Cochrane risk-of-bias tool for randomised trials (RoB 2)” [6].

## 3 Results

### 3.1 Description of the studies

The study selection process is shown in Supplementary Fig. 1. A total of 16 studies, published between 2010 and 2025 and encompassing 56 661 patients, were included after screening. Most were retrospective single-center cohorts, complemented by two large population-based database studies [7], [8] and one randomized controlled trial [9]. Definitions of IH varied widely, ranging from registry coding to clinical examination, imaging, or patient-reported outcomes. Considerable heterogeneity was observed in surgical specimen extraction sites (supraumbilical, infraumbilical, Pfannenstiel/suprapubic, midline, or off-midline) and in fascial closure techniques. Quality and RoB assessments are summarized in Supplementary Fig. 2. Nine (55%) studies presented a high or very high RoB, and only one had a low RoB [9].

### 3.2 Reported proportion and outcomes

The reported proportion of IH after MIRP ranged from 0.04% to 31%, reflecting heterogeneity in definitions and detection methods across studies (Table 1). When reported, the median time to diagnosis ranged from 4.7 mo to 5 yr, with a median reported value of approximately 14 mo across studies (available for 7/16 studies). The surgical repair proportion (available for 4/16 studies) ranged from 6.5% to 81% (total *n* = 348/1354), with an emergency repair proportion (available for 2/16 studies) ranging from 0.5% to 15% (total *n* = 12/262). Port-site hernia was infrequently reported, occurring in less than 1% of cases, and the scarcity and inconsistency of available data precluded meaningful pooled analysis (Supplementary Table 1).

**Table 1:** Study characteristics, incisional hernia proportions, and comparative results

**Author**	**Journal**	**Date**	***N***	**Experimental arm**	**Control arm**	**Primary end point**	**IH definition**	**Incisional Hernia (*N*, %)**	**Time to diagnosis**	**Port-site Hernia**
Peyrottes et al. [2]	European Urology Focus	2025	629	Transperitoneal RARP	NA	Proportion of IH	Systematic physical exam	76 (12.1%)	Proportion at 1 yr	NA
Honma et al. [15]	Hernia	2025	145	Transperitoneal RARP	NA	Proportion of IH	CT images	45 (31%)	Median time to diagnosis: 12 mo (range, 5–60 mo)	NA
Van Horn et al. [9]	Journal of Endourology	2025	1465 (1118 periumbilical vs 347 Pfannenstiel)	Pfannenstiel extraction of the specimen	Periumbilical extraction of the specimen	Proportion of IH	Symptoms, physical exam, and/or imaging	83 (7.4%) in the periumbilical group versus 2 (0.58%) in the Pfannenstiel group	Median time to diagnosis (periumbilical group only): 7.62 mo (IQR, 4.70–15.6 mo)	NA
Derekoylu et al. [10]	Journal of Laparoendoscopic & Advanced Surgical Techniques	2025	192 (135 periumbilical vs 57 Pfannenstiel)	Pfannenstiel extraction of the specimen	Periumbilical extraction of the specimen	Proportion of IH	NA	14 (10.4%) in the periumbilical group versus 2 (3.5%) in the Pfannenstiel group	NA	NA
Gaffney et al. [7]	Journal of Urology	2024	994 (482 transverse vs 512 vertical) a	Transverse supraumbilical incision	Vertical supraumbilical incision	Proportion of IH	Hierarchal predetermined criteria: 1. Physical exam 2. Self-reported hernia repair 1. 3. Self-reported symptoms	97 (19%) in the vertical group versus 100 (21%) in the transverse group	Proportion at 15 mo	NA
Timm et al. [17]	Journal of Robotic Surgery	2020	186	Transperitoneal RARP	NA	Proportion of IH	Physical exam or US images	16 (8.6%)	Mean time to diagnosis: 11.8 mo (IQR, 8–16 mo)	2 (1%)
Hermann et al. [5]	Scandinavian Journal of Urology	2017	19 743 (11 485 ORP vs 8258 MIRP)	MIRP	ORP	Proportion of IH	Declarative (register study)	1.4% in the ORP group versus 2.3% in the MIRP	Proportion at 5 yr	NA
Seveso et al. [13]	International Journal of Surgery	2017	800 (400 midline vs 400 off-midline)	Off-midline extraction of the prostate	Midline extraction of the prostate	Proportion of IH	Physical exam or CT images	5% in the midline group versus 4.5% in the off-midline group	Mean time to diagnosis: 20.2 mo (midline) versus 18.2 mo (off-midline)	NA
Fridriksson et al. [6]	Journal of Surgical Oncology	2017	19 712 (11 212 ORP vs 8500 RARP)	Transperitoneal RARP	ORP	Proportion of IH	Declarative (register study)	232 (2%) in the ORP group versus 191 (2.24%) in the RARP group	NA	NA
Chennamsetty et al. [18]	Advances in Urology	2015	577	Transperitoneal RARP	NA	Proportion of IH	Self-reported symptoms	48 (8.3%)	Median time to diagnosis: 1.2 yr (IQR, 0.5–2.9; range, .01–9.1)	2 (0.4%)
Carlsson et al. [8]	Journal of Urology	2013	9994 (6795 ORP vs 3199 MIRP)	MIRP	ORP	Proportion of IH	Declarative (register study)	100 (1.5%) in the ORP group versus 158 (4.9%) in the MIRP group	Proportion at 36 mo	NA
Liss et al. [11]	Urology	2013	1000 (735 vertical vs 265 transverse)	Transverse supraumbilical incision	Vertical supraumbilical incision	Proportion of IH	NA	40 (5.4%) in the vertical group versus 1 (0.4%) in the transverse group	NA	NA
Kang et al. [19]	Journal of Endourology	2012	498	Transperitoneal RARP	NA	Proportion of IH	Clinical examination on patient complaints	2 (0.04%)	Median time to diagnosis: 15 mo Median follow-up: 18 mo (range, 3–48 mo)	0 (0%)
Beck et al. [12]	Urology	2011	900 (735 vertical vs 165 transverse)	Transverse supraumbilical incision	Vertical supraumbilical incision	Proportion of IH	Physical exam or self-reported symptoms	5.3% in the vertical group versus 0.6% in the transverse group	Mean follow-up time: 3.6 yr (vertical group) and 1.5 yr (transverse group)	NA
Chalasani et al. [20]	Canadian Journal of Urology	2010	153	BMI >30	BMI <30	Proportion of IH	NA	3 (2%) in the BMI >30 group versus 2 (1.3%) in the BMI <30 group	NA	NA
Fuller et al. [14]	Journal of Endourology	2010	250	Transperitoneal RARP	NA	Proportion of IH	NA	10 (4%)	Mean time to diagnosis: 14.8 mo Mean follow-up: 35 mo	2 (0.8%)

RARP = Robot-Assisted Radical Prostatectomy; ORP = Open Retropubic Prostatectomy; MIRP = Minimally Invasive Radical Prostatectomy; IH = incisional hernia; CT = computed tomography; US = Ultrasound; BMI = body mass index; IQR = interquartile range; NA = not applicable.

a With data available for analysis.

### 3.3 Comparative outcomes

Five studies compared the reported proportion of IH according to specimen extraction strategy. Three large population-based studies compared open radical prostatectomy (ORP) with MIRP [7], [8], [10]. Across these studies, the reported proportion of IH was consistently lower following ORP than following MIRP, with reported proportions of 2.0% versus 2.2% [8], 1.5% versus 4.9% at 36 mo [10], and 1.4% versus 2.3% at 5 yr [7]. Two recent comparative cohort studies directly evaluated specimen extraction site during MIRP [11], [12]. Both reported lower proportions of IH following transverse suprapubic (Pfannenstiel) extraction than periumbilical extraction. In the larger study by Van Horn et al. [11], the reported proportion of IH was 0.58% following Pfannenstiel extraction compared with 7.4% following periumbilical extraction, with a median time to diagnosis of 7.6 mo in the periumbilical group. Similarly, Derekoylu et al. [12] reported proportions of 3.5% and 10%, respectively.

Three studies compared vertical versus transverse extensions of the camera port site for specimen extraction [9], [13], [14]. The randomized controlled trial by Gaffney et al. [9] found no statistically significant difference in the reported proportion of IH between vertical and transverse incisions. In contrast, the two earlier observational studies reported statistically significant lower proportions of IH with transverse compared with vertical incisions [13], [14]. Given the differences in study design and susceptibility to confounding, these findings should be considered inconclusive.

Seveso et al. [15] compared midline (camera port-site) and off-midline (lateral port-site) vertical extractions in 800 patients and similarly found no difference in the reported proportion of IH.

### 3.4 Factors associated with IH

Several studies explored associations between patient or surgical characteristics and the reported proportion of IH [2], [7], [10], [14], [15], [16], [17], [18]. Factors reported to be associated with IH in individual studies included advanced age, obesity, larger prostate volume, postoperative parietal abscess, and prior abdominal surgery. However, these findings should be interpreted cautiously because definitions of IH, analytical methods, adjustment strategies, and follow-up duration varied substantially across studies. Only one study reported an association between fascial closure technique and IH occurrence, favoring interrupted figure-of-eight sutures [18]. Readers are referred to Supplementary Table 1 for a detailed overview of these exploratory findings.

## 4 Discussion

This systematic review suggests that IH following MIRP is an under-recognized yet relatively frequent complication. Reported proportions varied widely, from near zero [13] up to 30% [16], reflecting heterogeneity in definitions, detection methods, and follow-up. Higher reported IH proportions were typically observed in studies employing systematic imaging for detection, whereas lower proportions corresponded to clinically apparent hernias only, highlighting the impact of outcome definition on reported proportion. Nevertheless, the burden of IH is clinically meaningful: repair proportions reached up to 80% [12], and emergency repair was required in up to 15% of cases [2], underlining the need for preventive strategies.

Interpretation of proportion estimates warrants caution. The included studies used heterogeneous ascertainment strategies ranging from administrative registry coding to clinical examination, imaging detection, or patient-reported outcomes. These approaches capture different clinical phenomena and may not be directly comparable. Radiologically detected or self-reported bulges may overestimate the burden of clinically relevant IH requiring intervention, whereas registry-based approaches may underestimate the true proportion due to undercoding or incomplete capture of events treated outside the index institution. Consequently, the range of reported proportions should be interpreted as reflecting methodological variability rather than a definitive estimate of epidemiologic risk.

The most consistent observation across comparative studies is the potential influence of specimen extraction site on the reported proportion of IH. Two observational studies directly comparing transverse suprapubic (Pfannenstiel) and periumbilical extraction during MIRP consistently reported lower proportions of IH with the suprapubic approach. In addition, three large registry-based studies comparing ORP with MIRP similarly reported lower proportions of IH following ORP, which generally involves a lower midline or suprapubic extraction.

In contrast, technical refinements aimed at reducing hernia proportions through modification of the aponeurotic incision appear insufficient. A randomized control trial comparing vertical versus transverse extensions of the supraumbilical camera port site demonstrated no significant difference in IH proportion [9]. Similarly, Seveso et al. [15] reported no difference between midline and off-midline vertical extractions. These findings further support that extraction-site location may be an important contributor to IH occurrence.

Beyond surgical technique, several patient- and procedure-related risk factors were consistently identified, including advanced age, having obesity, larger prostate volume, postoperative parietal abscess, and prior abdominal surgery, corroborating consensual guidelines [19]. Fascial closure may also play a role: one study reported a lower proportion of IH with interrupted figure-of-eight sutures compared with continuous closure, suggesting that meticulous attention to closure technique and duration may help mitigate risk [18].

Several limitations should be acknowledged. First, the available evidence is largely retrospective, with more than half of the studies having a high or very high RoB. Retrospective study designs are particularly susceptible to selection bias, incomplete outcome ascertainment, and residual confounding due to nonrandom allocation of surgical techniques. These limitations may have affected both the likelihood of detecting IH events and the magnitude of the observed associations between surgical approaches and hernia risk. Second, several factors likely contributed to the substantial heterogeneity observed across studies, including differences in outcome ascertainment (registry coding, clinical examination, imaging, or patient-reported outcomes), duration of follow-up, study design (single-center cohorts versus population-based registries), surgical era, and surgeon-specific preferences regarding specimen extraction and fascial closure. These methodological and clinical differences likely account for a substantial proportion of the variability in reported proportion estimates. An additional limitation relates to the heterogeneous duration of follow-up across studies. Since IH is a time-dependent outcome, cumulative event proportions measured at different time points are not directly comparable. These limitations underscore the need for well-designed prospective studies with standardized definitions and systematic follow-up to better characterize the true proportion, risk factors, and preventive strategies for IH after RP.

From an evidence-certainty perspective, interpretation of the available comparative evidence is limited by several factors, including the predominance of observational studies with moderate-to-high RoB, substantial heterogeneity in outcome ascertainment and follow-up duration, indirect comparisons across different surgical approaches, and the small number of available comparative studies resulting in imprecise estimates. These limitations reduce confidence in the magnitude of the observed associations and underscore the need for prospective studies using standardized outcome definitions and follow-up protocols.

## 5 Conclusion

Taken together, these findings highlight that, although the true epidemiologic proportion of IH after MIRP remains difficult to quantify due to heterogeneous definitions and detection methods, the extraction site may be associated with reported IH occurrence and warrants further prospective evaluation.

  ***Author contributions***: Arthur Peyrottes had full access to all the data in the study and takes responsibility for the integrity of the data and the accuracy of the data analysis.

  *Study concept and design:* Peyrottes, Masson-Lecomte, Oberlin, Ploussard.

*Acquisition of data*: Peyrottes, Panthier, Dariane, Baboudjian.

*Analysis and interpretation of data*: Peyrottes, Ploussard, Masson-Lecomte.

*Drafting of the manuscript*: Peyrottes, Oberlin.

*Critical revision of the manuscript for important intellectual content*: Ploussard, Desgrandchamps, Mongiat-Artus.

*Statistical analysis:* Peyrottes.

*Obtaining funding:* None.

*Administrative, technical, or material support:* None.

*Supervision:* Ploussard, Dariane, Panthier, Masson-Lecomte, Desgrandchamps, Mongiat-Artus.

*Other:* None.

  ***Financial disclosures***: Arthur Peyrottes certifies that all conflicts of interest, including specific financial interests and relationships and affiliations relevant to the subject matter or materials discussed in the manuscript (eg, employment/affiliation, grants or funding, consultancies, honoraria, stock ownership or options, expert testimony, royalties, or patents filed, received, or pending), are the following: None.

  ***Funding/Support and role of the sponsor*:** None.

## Appendix A Supplementary material

Supplementary Data 1 PRISMA flow diagram, risk-of-Bias traffic-light and summary plots, and forest plot comparing the vertical versus transverse extension for the extraction of the prostate.
