# Supplementary material for: Incisional Hernia After Minimally Invasive Radical Prostatectomy: A Systematic Review
Source: Eur Urol Open Sci. 2026 Sep 28;93:42–7. doi: 10.1016/j.euros.2026.08.002 (PMC13652846; doi:10.1016/j.euros.2026.08.002)
Supplement: Supplementary file 1 [file mmc1.docx]

**Identification of studies via databases and registers**

Duplicate records removed

(n = 33)

Records identified: 298

Embase (n = 115)

PubMed (n = 35)

Web of Science (n = 148)

**Identification**

Records screened

(n = 265)

Records excluded

(n = 229)

Reports sought for retrieval

(n = 36)

Reports not retrieved

(n = 0)

**Screening**

Reports excluded: n = 20

Congress abstract (n = 4)

Data not informative (n = 4)

Editorial (n = 2)

Open approach only (n = 1)

Poster (n = 3)

Review (n = 3)

Single port only (n = 3)

Reports assessed for eligibility

(n = 36)

Studies included in review

(n = 16)

**Included**

**Supplementary Figure 1**: PRISMA flow diagram.
