# Supplementary material for: Incisional Hernia After Minimally Invasive Radical Prostatectomy: A Systematic Review
Source: Eur Urol Open Sci. 2026 Sep 28;93:42–7. doi: 10.1016/j.euros.2026.08.002 (PMC13652846; doi:10.1016/j.euros.2026.08.002)
Supplement: Supplementary file 2 [file mmc2.docx]

**A**

**B**

**Supplementary Figure 2**: Risk-of-Bias traffic-light and summary plots using A. the Risk Of Bias In Non-randomized Studies - of Interventions (ROBINS-I) tool, and B. the Revised Cochrane risk-of-bias tool for randomised trials (RoB 2) tool.
